# ‘How Long Do I Have?’ – Examining survival outcomes in laryngeal cancer patients managed with non‐curative intent in Northern UK: Insights from the Northern Head & Neck Cancer Alliance Retrospective Study

**DOI:** 10.1111/coa.14260

**Published:** 2024-12-11

**Authors:** Amarkumar Rajgor, Rhona Hurley, Catriona M. Douglas, Claire Paterson, James Moor, Shane Lester, Sara Sionis, Katharine Davies, James O'Hara, Gareth Inman, Terry Jones, David Winston Hamilton, Northern Head & Neck Alliance, Sarah Hill, Seamus O’Neill, Alison McLoughlin, Jemy Jose, Winson Wong, Michael Ho, Debbie Horne, Jarrod Homer, Matthew Kennedy, Emma Kinloch, Robert Metcalf, Iain Varley, Thomas Carroll, Sarah Healy, Helen Cocks, Michael Nugent, Leo Vassilou, Panos Kyzas, John Greenman, Andrew Schache, Jason Fleming, Joanne Patterson, Keith Hunter, Paula Parvulescu, Rachel Brooker, Richard Shaw, Stephanie Meysner, Ola Rominiyi, Olena Mandrik

**Affiliations:** ^1^ National Institute for Health & Care Research Doctoral Fellow in Otolaryngology, Population Health Sciences Institute Newcastle University Newcastle‐Upon‐Tyne UK; ^2^ Newcastle upon Tyne Hospitals NHS Foundation Trust Freeman Hospital Newcastle‐Upon‐Tyne UK; ^3^ NHS Greater Glasgow & Clyde Newcastle‐Upon‐Tyne UK; ^4^ Glasgow Head and Neck Research (GLAHNC) Group Glasgow UK; ^5^ CRUK Scotland Institute and School of Cancer Sciences, College of Medical and Veterinary Sciences University of Glasgow Glasgow UK; ^6^ Leeds Teaching Hospitals NHS Foundation Trust Leeds UK; ^7^ South Tees Hospitals NHS Foundation Trust Middlesbrough UK; ^8^ Sheffield Teaching Hospitals NHS Foundation Trust Sheffield UK; ^9^ Liverpool University Hospitals NHS Foundation Trust Liverpool UK; ^10^ Liverpool Head & Neck Centre Liverpool UK

**Keywords:** Laryngeal cancer, Multidisciplinary approach, Palliative care, Survival outcomes

## Abstract

**Introduction:**

Historically, 15% of laryngeal cancer patients undergo non‐curative management, but pragmatic data on this group are limited. This information is crucial to help patients make informed decisions about their care. Supported by the Northern Head & Neck Alliance, this retrospective study is the first to present survival outcomes for non‐curative laryngeal cancer patients in Northern UK.

**Methods:**

Retrospective data were compiled for patients with laryngeal squamous cell cancer from five large tertiary head and neck centres in Northern UK (Newcastle, Glasgow, Sheffield, Leeds, and Middlesbrough). The collected data encompassed demographic details, treatment and clinical outcomes.

**Results:**

Among 373 patients, the mean age was 72, and 73% were male. The median follow‐up was 6 months. 17% had early‐stage (T1‐2), and 83% had late‐stage (T3‐4) disease. By data collection, 99% had died.

The mean survival time (MST) was 9.1 months. Patients with metastases had an MST of 6.9 months, while those without had 9.4 months. Early‐stage patients had an MST of 13.3 months, compared to 8.2 months for advanced disease. By subsite, MSTs were 8.2 months for supraglottic, 12.5 for glottic, 5.5 for subglottic, and 7.9 for transglottic cancers.

**Conclusion:**

This study stands as the first to explore survival outcomes in laryngeal cancer patients undergoing non‐curative management. The findings can provide valuable insights for informing patients about survival in the absence of radical treatment, facilitating important decision‐making conversations.

**Summary**

- This study represents the first investigation to provide real‐life pragmatic data on patients undergoing non‐curative management for laryngeal cancer, including a detailed breakdown of survival times stratified by tumour stage, anatomical subsite, and metastases at presentation.
- The mean survival time (MST) for all laryngeal cancers was 9.1 months.
- Patients with metastases had a MST of 6.9 months, while those without had 9.4 months.
- Early‐stage patients had an MST of 13.3 months, compared to 8.2 months for advanced disease.
- By subsite, MSTs were 8.2 months for supraglottic, 12.5 for glottic and 7.9 for transglottic cancers.

## 1 Introduction

Head and neck cancer stands as the seventh most prevalent cancer worldwide, with 660 000 new cases and 325 000 deaths attributed to the disease annually [1, 2]. A significant number of these cases occur in the larynx, affecting 2400 patients in the United Kingdom (UK) each year, and affects disproportionately those from socioeconomically disadvantaged backgrounds [3]. Additionally, these patients often suffer from multiple comorbidities that share the common risk factors associated with laryngeal cancer, including alcohol consumption and smoking, further compromising their overall health [4]. While early‐stage disease offers an 89% three‐year survival rate, this figure drops significantly to around 50% in advanced disease [5, 6]. Furthermore, approximately half of all patients receive a diagnosis at an advanced stage, resulting in poor survival [5].

Given this high morbidity and the prevalence of advanced disease, 15%–20% of patients receive non‐curative intent care at the outset [7]. This approach is adopted for patients either unsuitable for aggressive treatment or with incurable disease [8]. Consequently, the multi‐disciplinary team is often confronted with a challenging question from patients:

“How long do I have if I don't get treatment to cure my cancer?”

Qualitative research with head and neck cancer patients has shown that almost all patients with incurable disease are interested in understanding their life expectancy [9, 10]. Despite this common scenario, there is a lack of comprehensive data on the survival outcomes of laryngeal cancer patients undergoing non‐curative management. Therefore, clinicians often rely on anecdotal experience when estimating survival in this context. However, this information is needed to facilitate informed decision‐making conversations in collaboration with patients and empower them during their final stages of life. To effectively address such research questions, the Northern Head & Neck Alliance was established in 2022 under the auspices of the Northern Health Sciences Alliance. This coalition consists of nine major head and neck cancer centres in Northern England and Scotland, committed to collaborative research efforts.

### 1.1 Objective

This retrospective, collaborative study will be the first to provide insights into the survival outcomes of patients with laryngeal cancer who are undergoing non‐curative management in Northern UK.

## 2 Methods

### 2.1 Patient Population

Retrospective patient data spanning a 6‐year period from January 2015 to December 2021 were collated from five major head and neck specialist centres across Northern United Kingdom. These centres comprised Greater Glasgow & Clyde Hospitals, Leeds Teaching Hospitals NHS Trust, Newcastle Upon Tyne NHS Foundation Trust, Sheffield Teaching Hospital and South Tees Hospitals NHS Foundation Trust. Inclusion criteria for this study comprised of patients diagnosed with biopsy proven squamous cell laryngeal carcinoma who underwent non‐curative intent management (*n = 373*). Of note, this study is a secondary analysis of a larger data collection effort across the NHNA which collated data on 2401 patients across Northern UK with laryngeal cancer.

The collected data included patient demographics, American Joint Committee on Cancer (AJCC) Tumour Node Metastases staging, anatomical subsite of the primary malignancy, and follow‐up data. Patients lacking sufficient follow‐up or medical data were excluded.

### 2.2 Non‐curative Intent Management

The palliative and best supportive care approach was provided to patients who were unfit for aggressive treatment or had incurable disease. This patient group received holistic management aimed at enhancing or maintaining their quality of life. This care was overseen by a multidisciplinary team and for some, involved administering a short course of radiotherapy or chemotherapy for symptom management.

### 2.3 Statistical Analysis

Statistical analyses were conducted utilising SPSS version 29.0.1.1. Kaplan–Meier survival analyses were employed to provide a comprehensive assessment of survival (including estimated overall survival (OS) and disease‐specific survival (DSS) rates).

## 3 Results

### 3.1 Patient Characteristics

The study cohort consisted of 373 patients who received non‐curative care for laryngeal cancer. The mean age of the patients was 72.0 years, with a notable male predominance (73%). The median follow‐up time was 6 months. Early‐stage disease (T1‐2) was observed in 17% of patients (*n* = 65), while the majority (83%) had late‐stage disease (T3‐4). At the time of data collection, 99% of patients (*n* = 368) had deceased. Further detail is shown in Tables 1 and 2 including missing data.

**TABLE 1:** Patient characteristics of cohort.

	*n* (%)
Patients included	
Total (*n*)	373
Glasgow & Clyde Hospitals NHS Trust	196 (53%)
Leeds Teaching Hospitals NHS Trust	41 (11%)
Newcastle Upon Tyne NHS Trust	18 (5%)
South Tees Hospitals NHS Trust	58 (16%)
Sheffield Teaching Hospitals	60 (16%)
Baseline patient demographics	
Age^a^	
Age (mean ± SD)	72.0 ± 11.7
Gender^a^	
Male	230 (73%)
Female	85 (27%)
Smoking status^b^	
Non‐smoker	12 (6%)
Ex‐smoker	72 (33%)
Current smoker	133 (62%)
Tumour subsite	
Subglottis	9 (2%)
Glottis	84 (23%)
Supraglottic	215 (58%)
Transglottic	46 (12%)
Indeterminate	19 (5%)
American Joint Committee on Cancer	
TNM staging	
T‐stage	
T1	19 (5%)
T2	46 (12%)
T3	123 (33%)
T4	185 (50%)
N‐stage	
N0	190 (51%)
N1	36 (10%)
N2	121 (32%)
N3	26 (7%)
M‐stage	
M0	337 (90%)
M1	36 (10%)

*Note: n* (%): indicates the number of patients, with the percentage relative to the total cohort shown in parentheses.

^a^ Missing data: information on age and gender is unavailable for 58 patients.

^b^ Missing data: smoking status data is unavailable for 156 patients.

**TABLE 2:** Follow‐up data on patient cohort.

	*n* (%)
Follow‐up data	
Median follow‐up (Range)	6 months (0–128)
Mean survival time (Months)	9.3 months
Overall mortality rate	
Alive	5 (1%)
Deceased	368 (98.7%)
Disease‐specific mortality^a^	
Alive	4 (1%)
Disease‐associated death	289 (76%)
Death unrelated to disease	22 (7%)

*Note: n* (%): Indicates the number of patients, with the percentage relative to the total cohort shown in parentheses.

^a^ Missing data: Information on exact cause of death is unavailable for 58 patients.

### 3.2 Mean Survival Time

In the entire cohort (*n* = 373), the mean survival time was 9.1 months, while the median survival was 6 months. Table 3 provides further detail on mean survival times, along with their associated standard deviations and standard errors. It stratifies the data by various factors, including the presence of metastases, AJCC (8th Edition) T‐stage (early vs. advanced) and anatomical subsite of the cancer.

**TABLE 3:** Mean survival time in patients with laryngeal cancer managed with non‐curative intent.

	*n*	Mean Survival Time (± SD)	SE
Entire Cohort	373	9.1 months (±8.4)	0.4
Presence Of Metastases			
Metastases At Presentation	36	6.9 months (± 6.1)	1.6
No Metastases	337	9.4 months (±8.8)	0.6
Tumour Stage (AJCC 8th Edition)			
Early Disease (T1‐2)	65	13.3 months (± 9.2)	1.7
Advanced Disease (T3‐T4)	308	8.2 months (±7.1)	0.6
Anatomical Subsite Of Laryngeal Cancer			
Supraglottic	215	8.2 months (± 8.1)	0.6
Glottic	84	12.5 months (± 12.3)	1.5
Subglottic	9	5.5 months (±5.4)	1.2
Transglottic	46	7.9 months (±7.8)	1.7

Abbreviations: SE = Standard Error, SD = Standard Deviation.

### 3.3 Survival Probability Outcomes

**FIGURE 1:**
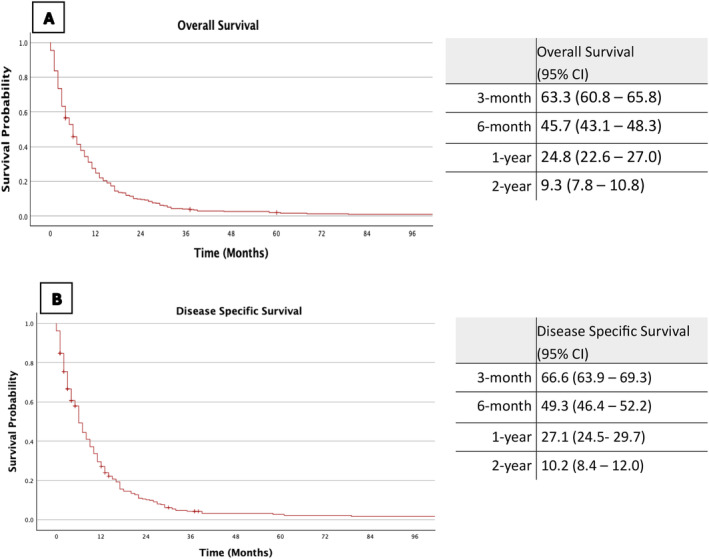
Kaplan Meier Survival Curves with associated estimated survival rates for entire cohort. [A] Overall Survival: Kaplan Meier survival curve with associated estimated overall survival rates for entire cohort. [B] Disease Specific Survival: Kaplan Meier survival curve with associated estimated disease‐specific survival rates for entire cohort.

The estimated 3‐month, 6‐month, 1‐year and 2‐year overall survival (OS) rates were 63.3%, 45.7%, 24.8% and 9.3%, respectively (Figure 1A). Disease‐specific survival (DSS) rates for the same intervals were 66.6%, 49.3%, 27.1% and 10.2%, respectively (Figure 1B).

The estimated 1‐year OS rate for patients with distant metastases was 17.8% (95% CI: 11.3–24.3), compared to 25.2% (95% CI: 22.8–27.6) for those without metastases. The estimated 1‐year DSS rates were comparable at 18.7% (95% CI 11.3–26.1) and 27.6% (95% CI 24.9–30.3) for M1 and M0 disease, respectively.

**FIGURE 2:**
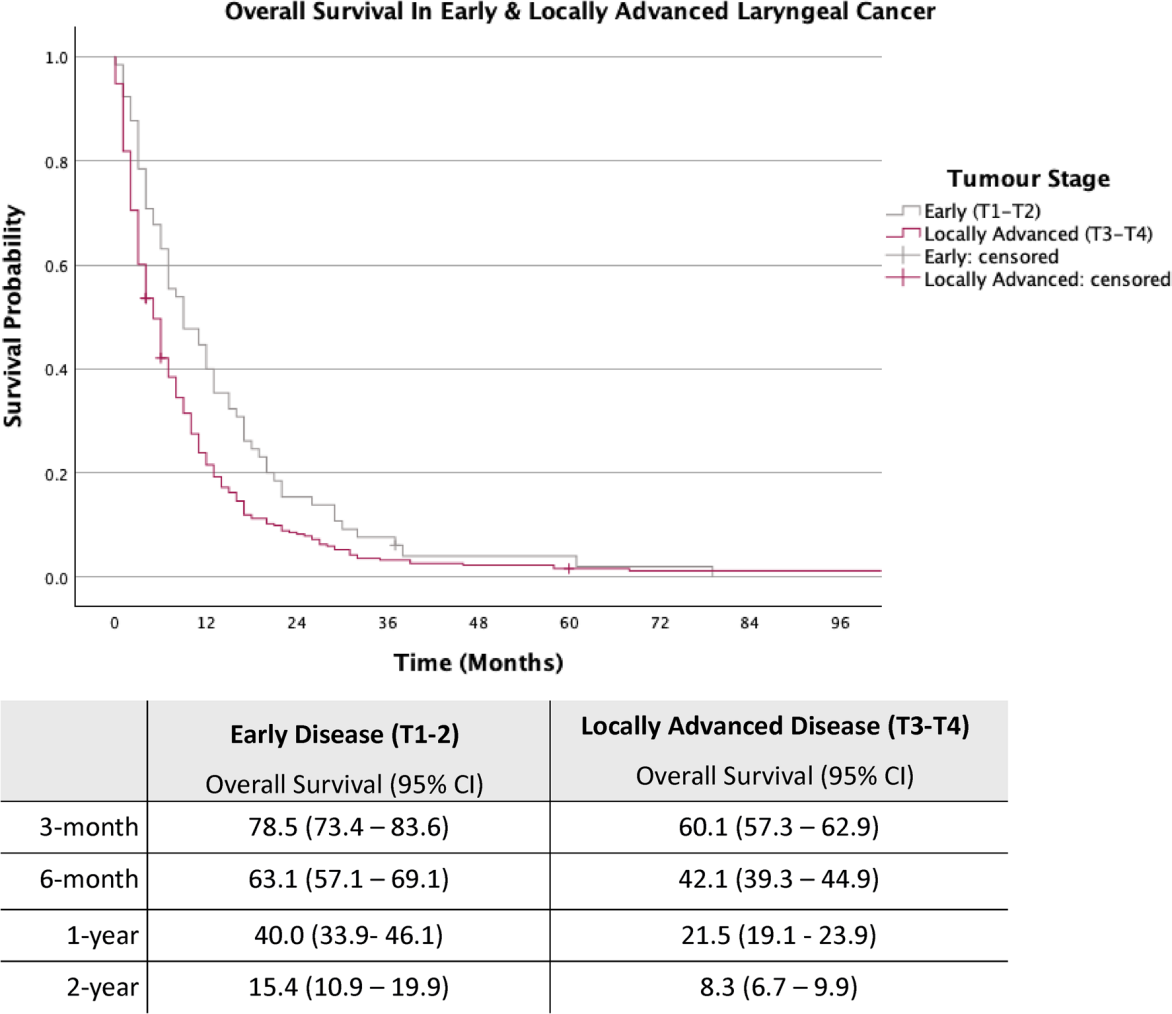
Kaplan–Meier survival curves with associated estimated overall survival rates for patients with early and locally advanced laryngeal cancer.

**FIGURE 3:**
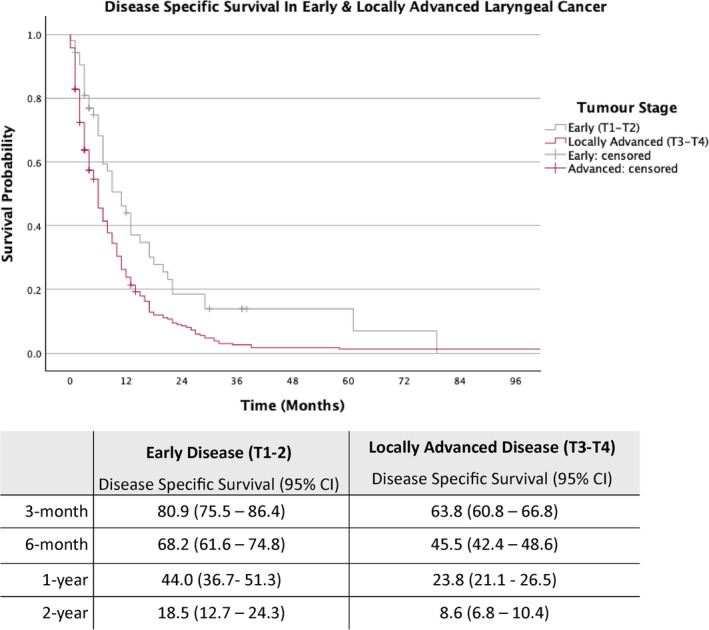
Kaplan–Meier survival curves with associated estimated disease‐specific survival rates for patients with early and locally advanced laryngeal cancer.

The cohort was further stratified into early laryngeal cancer (T1‐T2) and locally advanced laryngeal cancer (T3‐T4) (Figures 2 and 3).

As depicted in Figure 2, for patients with early‐stage disease, the estimated OS rates at 3‐month, 6‐month, 1‐year and 2‐year were 78.5%, 63.1%, 40.0% and 15.4%, respectively, while those with locally advanced disease (T3‐T4), the estimated OS rates for the same intervals were 60.1%, 42.1%, 21.5% and 8.3%.

As demonstrated in Figure 3, for patients with early‐stage disease, the estimated DSS rates at 3‐month, 6‐month, 1‐year and 2‐year were 80.9%, 68.2%, 44.0% and 18.5%, respectively, while those with locally advanced disease (T3‐T4), the estimated DSS rates for the same intervals were 63.8%, 45.5%, 23.8% and 8.6%.

## 4 Discussion

To date, this study represents the first investigation to provide real‐life pragmatic data on patients undergoing non‐curative management for laryngeal cancer, including a detailed breakdown of survival times stratified by tumour stage, anatomical subsite and metastases at presentation. Several important findings emerge, which could significantly aid informed discussions with patients.

Based on these results, we feel a number of recommendations can be made in our region. Firstly, on counselling patients with regard to prognosis, the average survival time is approximately nine months. Depending on the individual patient circumstances further detail can be provided. Patients with metastases at presentation have a mean survival of 6.9 months, while those without have 9.4 months. However it is important to highlight that the number of patients with metastases is small (*n = 36*), and there is overlap in the standard deviations between those with and without metastases. Thus, we should be cautious about overstating the difference. However, this indicates that poor survival rates are mainly due to advanced disease or patients being unfit for treatment.

Early‐stage (T1‐T2) patients have a mean survival of 13.3 months, compared to 8.2 months for advanced‐stage (T3‐T4) patients. By anatomical subsite, the mean survival times are 8.2 months for supraglottic, 12.5 months for glottic and 7.9 months for transglottic cancer patients. Similar to patients with or without metastases, patients with subglottic disease should be considered carefully since the number of patients in this subgroup is also small (*n = 9*). Furthermore, the estimated 1‐year OS probability for all laryngeal cancer patients managed non‐curatively is 24.8%. In patients with early laryngeal disease, the estimated 1‐year OS rate is higher at 40%, while those with locally advanced disease have an estimated 1‐year OS rate of 21.5%.

Laryngeal cancer, alongside hypopharyngeal cancer, presents the poorest survival outcomes among head and neck cancers [11]. Therefore, dedicated studies focusing on these cancers are essential. While prior studies have not delved into the disease trajectory of laryngeal cancer patients managed with non‐curative intent, various studies have explored head and neck cancers collectively. For example, Mayland et al. assessed the disease trajectory in all head and neck cancers managed with non‐curative intent from the ‘Head and Neck 5000’ clinical cohort study [12]. This demonstrated that around two‐thirds of patients were deceased within 12 months, attributing a poorer prognosis to factors like increasing age, multiple comorbidities, advanced disease and cancers affecting the larynx. Our study reveals even worse survival outcomes, with approximately 75% of patients deceased by 12 months. This disparity could be attributed to several factors, including the exclusive inclusion of laryngeal cancers in our study, which typically exhibit worse survival than other head and neck cancers. Additionally, despite regional health disparities, particularly in the North of England where mortality and morbidity rates are generally higher, the similar OS and DSS rates in our results suggest that the poor outcomes are likely due to cancer‐related deaths rather than comorbidities [13].

Despite the limited literature on this subject, a study conducted in the US demonstrated patients receiving palliative treatment were more likely to be older ( > 65 years), of Afro‐Caribbean ethnicity, have late‐stage disease or have hypopharyngeal cancer [14]. In our cohort, the patients also tended to be older and have advanced disease. With regard to survival time from diagnosis, the literature suggests ranges from five to eight months which is comparable to our results [10, 14, 15]. It is important to note that a number of patients lived beyond 18 months despite being given palliative care at the outset. This also underscores the critical role of high‐quality palliative and supportive care for individuals facing a terminal diagnosis, in particular the use of chemotherapy and radiotherapy for suitable patients.

Survival data can offer valuable guidance for patients and their families. A qualitative pilot study with head and neck cancer patients highlighted that nearly all patients with incurable disease express interest in understanding their life expectancy [10]. Their primary reasons include grappling with uncertainty, desiring to spend meaningful time with their families, and considering potential plans for the near future. For laryngeal cancer, this study provides an opportunity to empower patients by offering evidence‐based survival timelines, thus supporting them in making informed decisions in these extremely difficult circumstances.

This study by the NHNA marks the first evaluation of survival outcomes for laryngeal cancer patients managed without radical treatment. It includes data from five diverse head and neck cancer centres across Northern UK, representing various geographical regions. The study addresses a crucial patient‐centred question, offering a comprehensive, real‐world understanding of survival outcomes for laryngeal cancer patients receiving non‐curative management in the UK. These findings offer robust evidence to inform and support patients navigating these challenging circumstances.

### 4.1 Strengths, Limitations

A major strength of this study is that it is the first to evaluate survival outcomes exclusively in laryngeal cancer within a non‐curative context. This allows better generalisability for this complex patient group. Furthermore, this is one of the largest studies (*n* = 373) in the literature evaluating such outcomes in any head and neck cancer.

One of the key limitations of this study includes the lack of a detailed breakdown of palliative approaches for all patients, such as specific surgical, chemotherapy, or radiotherapy interventions.

Furthermore, as highlighted earlier, several sub‐groups, including those with metastases and subglottic disease, had small patient numbers. Therefore, we should be cautious in interpreting and applying these results. Nonetheless, overall, this data presents real‐life pragmatic data on survival in this complex group of patients.

## 5 Conclusion

This study stands as the only exploring survival outcomes exclusively in laryngeal cancer patients undergoing non‐curative care. The findings can potentially provide valuable insights for informing patients about survival in the absence of radical treatment, facilitating important decision‐making conversations. The results are anticipated to be beneficial for both clinicians and patients alike.

## Author Contributions

Amarkumar Rajgor, David Winston Hamilton, and Terry Jones, with contributions from James O'Hara, designed the study. The core writing committee and key data collators consisted of Amarkumar Rajgor, Rhona Hurley, Catriona M. Douglas, Claire Paterson, James Moor, Shane Lester, Sara Sionis, Katharine Davies, James O'Hara, Gareth Inman, Terry Jones and David Winston Hamilton. All authors including collaborators across the NHNA reviewed and provided critical revisions to the manuscript.

## Ethics Statement

No ethical approval was required after evaluation using the Health Research Authority decision tool (available at: http://www.hra‐decisiontools.org.uk/research/).

## Consent

The authors have nothing to report.

## Conflicts of Interest

The authors declare no conflicts of interest.

### Peer Review

The peer review history for this article is available at https://www.webofscience.com/api/gateway/wos/peer‐review/10.1111/coa.14260.

## Data Availability

Data are available on request from authors.

## Authorship Statement & Participation (Northern Head & Neck Alliance)

**Writing committee & key data collators:** Amarkumar Rajgor, Rhona Hurley, Catriona M Douglas, Claire Paterson, James Moor, Shane Lester, Sara Sionis, Katharine Davies, James O'Hara, Gareth Inman, Terry Jones, David Winston Hamilton.

**Project Management:** Sarah Hill (The NHSA—Project Manager), Seamus O'Neill (The NHSA—Chief Executive).

**Additional collaborators across Northern Head & Neck Alliance:** Alison McLoughlin (East Lancashire Hospitals NHS FT); Jemy Jose, Winson Wong (Hull University Teaching Hospitals NHS FT); Michael Ho (Leeds Teaching Hospitals NHS FT); Debbie Horne (Liverpool Head & Neck Cancer Centre); (Liverpool University Hospitals NHS FT); Jarrod Homer (Manchester University NHS FT); Matthew Kennedy (Newcastle upon Tyne Hospitals NHS Foundation Trust (NUTH)); Emma Kinloch, Robert Metcalf (Salivary Gland Cancer UK); Iain Varley (Sheffield Childrens Hospital NHS FT); Thomas Carroll (Sheffield Teaching Hospitals NHS FT); Sarah Healy (South Tees Hospitals NHS FT); Helen Cocks, Michael Nugent (Sunderland & South Tyneside NHS FT); Leo Vassilou, Panos Kyzas (University of Central Lancashire (UCLan)); David Conway (University of Glasgow); John Greenman (University of Hull); Andrew Schache, Jason Fleming, Joanne Patterson, Keith Hunter, Paula Parvulescu, Rachel Brooker, Richard Shaw, Stephanie Meysner (University of Liverpool & Liverpool University Hospitals NHS FT); Ola Rominiyi, Olena Mandrik (University of Sheffield).
